# p53 Antibodies as a Diagnostic Marker for Cancer: A Meta-Analysis

**DOI:** 10.3390/molecules26206215

**Published:** 2021-10-14

**Authors:** Navid Sobhani, Giandomenico Roviello, Alberto D’Angelo, Raheleh Roudi, Praveen Kumar Neeli, Daniele Generali

**Affiliations:** 1 Department of Medicine, Section of Epidemiology and Population Sciences, Baylor College of Medicine, Houston, TX 77030, USA; praveenkumar.neeli@bcm.edu; 2 Department of Health Sciences, University of Florence, 50121 Florence, Italy; giandomenico.roviello@unifi.it; 3 Department of Biology and Biochemistry, University of Bath, Bath BA2 7AY, UK; ada43@bath.ac.uk; 4 Department of Medicine, University of Minnesota Medical School, Minneapolis, MN 55455, USA; roudi002@umn.edu; 5 Department of Medical, Surgical and Health Sciences, University of Trieste, Cattinara Hospital, Strada Di Fiume 447, 34149 Trieste, Italy

**Keywords:** meta-analysis, p53, serum p53 antibodies, cancer survival prognostic biomarker

## Abstract

**Importance**: The protein p53 is an unequivocal tumor suppressor that is altered in half of all cancers. The immune system produces systemic p53 autoantibodies (p53 Abs) in many cancer patients. **Objective**: This systemic review and meta-analysis focuses on the prognostic value of p53 Abs expressed in the serum of patients with solid tumors. **Data Sources**: All the clinical investigations were searched on PubMed from the first study dated 1993 until May 2021 (date of submission of the manuscript). **Study Selection**: Studies were included that met the following criteria: (1) participants with cancer; (2) outcome results expressed in relation to the presence of a p53 antibody; (3) a primary outcome (disease-free survival, overall survival or progression-free survival) expressed as hazard ratio (HR). The following exclusion criteria were used: (1) insufficient data available to evaluate outcomes; (2) animal studies; (3) studies with less than 10 participants. As a result, 12 studies were included in the analysis. **Data Extraction and Synthesis**: PRISMA guidelines were used for abstracting and assessing data quality and validity by three independent observers. The summary estimates were generated using a fixed-effect model (Mantel–Haenszel method) or a random-effect model (DerSimonian–Laird method), depending on the absence or presence of heterogeneity (I^2^). **Main Outcome(s) and Measure(s)**: The primary study outcome was to determine the prognostic value of p53 Abs from a large population of patients with solid tumors, as determined before data collection. **Results:** In total, 12 clinical studies involving 2094 patients were included in the meta-analysis, and it was determined that p53 Abs expression in the serum significantly correlated with poorer survival outcomes of cancer patients (95% CI 1.48 [1.24, 1.77]; *p* < 0.00001). **Conclusions and Relevance:** This is the first meta-analysis proving the diagnostic utility of p53-Abs for cancer patients in predicting poorer outcomes. The serum-p53 value (s-p53-value) may be useful for future theranostics.

## 1. Introduction

The P53 protein is an unequivocal tumor suppressor mutated in almost half of human cancers [1,2,3,4]. It is autoregulated by MDM2, an E3 ubiquitin ligase [5,6].

Mice lacking MDM2 show embryonic lethality, while the dual presence of p53 and MDM2 can rescue lethality [7]. The p53 mutation in cancer (p53-mut) does not activate the expression of the E3 ligase. Consequently, degradation of p53 protein is not downmodulated [8]. High expression of p53 by cells recapitulates in T-cells the production of antibodies against mutant or wild type p53 [8]. On the other hand, in many cancer patients the p53-wt region is exposed and serum antibodies are generated against p53-wt. These can be detected by ELISA method. The roles of these antibodies are not yet clearly understood.

Prognostic biomarkers have a crucial role in measuring the progression of diseases from samples of patients, such as metastasis in cancer, and they can aid clinicians in intervening with more precise medical interventions. In addition to the common theory that in humans the loss of p53 increases genomic instability, this loss has been linked to the proliferation of the stem-cell characteristic that ultimately leads to highly aggressive cancers with invasive and metastatic properties. p53 antibodies (s-p53-Abs) are stably expressed in the sera of cancer patients, and could have an important prognostic application. Many clinical studies have assessed in cancer patients the correlation between the expression of s-p53-Abs with tumor invasiveness grades, metastasis and prognosis [9].

In our review, we performed a meta-analysis of the current literature, investigating the prognostic role of serum p53-Abs in cancer patients.

## 2. Results

**Figure 1:**
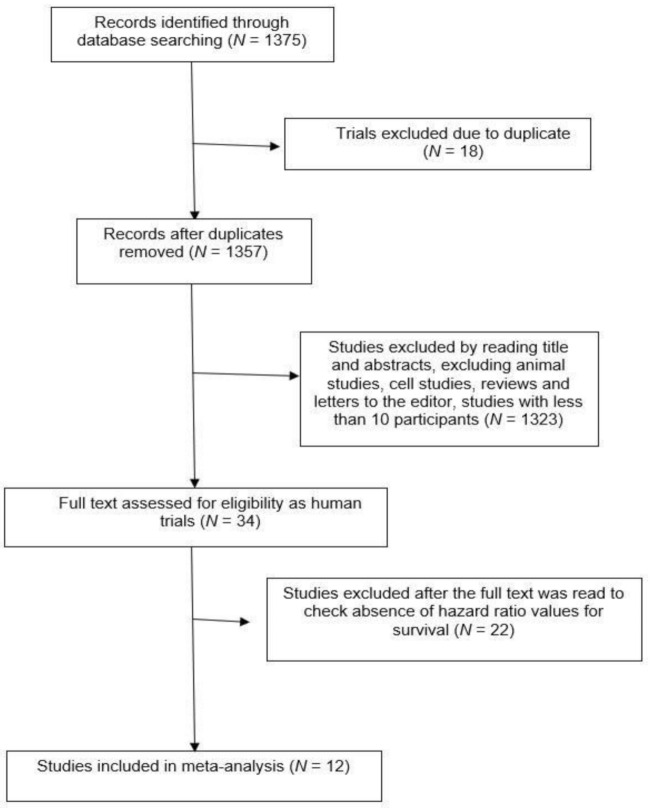
Flowchart of literature research strategy.

**Figure 2:**
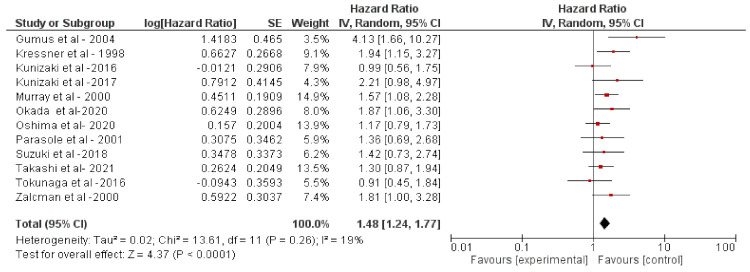
Meta-analysis of serum p53-antibodies. The prognostic value of p53 antibodies in the sera of cancer patients from eight clinical investigations was investigated in this meta-analysis.

After screening the article according to flow chart in Figure 1, 12 studies were selected [10,11,12,13,14,15,16,17,18,19,20,21]. A total of 2094 patients were included from these studies. The solid cancer patients were treated with adjuvant chemotherapy (such as cyclophosphamide, docetaxel, fluorouracil, epirubicin, methotrexate, and vinorelbine), anti-HER2 (trastuzumab, pertuzumab or lapatinib), endocrine therapy (such as goserelin, and tamoxifen), or combined treatment with Herceptin, chemotherapy, and the nonsteroidal anti-inflammatory drug celecoxib, also including radiotherapy or a surgical component in some cases (Table 1 and Table 2). The pooled analysis revealed that s-p53-Abs is a negative prognostic factor (HR: 148 [1.24, 1.77]; *p* < 0.0001, Figure 2) in cancers. The analysis was performed using a random-effects model (accounting for effect size heterogeneity; I^2^ = 19%).

**Figure 3:**
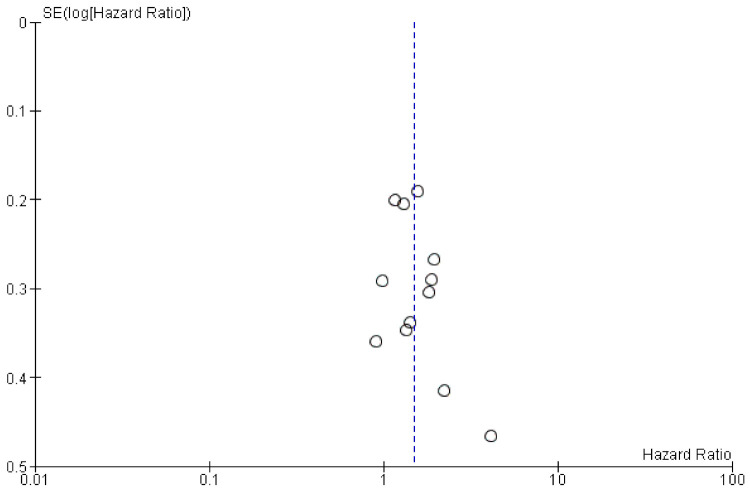
The funnel plot of included studies.

The funnel plot (Figure 3) of the included studies showed a symmetric funnel plot and no significant publication bias was identified.

## 3. Discussion

The meta-analysis showed that high levels of p53 antibodies significantly correlated with worse clinical outcomes. However, our study had some limitations. First, the retrospective nature of the study was intrinsically susceptible to biases. Second, different forms of solid tumors were included pre- or post-treatment with various types of therapies, as the typology requirements were at different stages. Consequently, in our analysis patients were observed independently of treatment and tumor type because of the relatively low number of randomized studies at our disposal. Third, there was a lack of follow-up with patients from different clinical trials. Thus, differences in survival probability may have been influenced by the durations of the studies. This may have given rise to different age populations, which could ultimately have affected the data. All these variables may ultimately have influenced the results.

As medicine advances, studies involving greater numbers of patients could help to evaluate the impact of our findings and treatment response.

In summary, p53 is a well-established tumor suppressor, and its absence is commonly found in patients diagnosed with cancer. The p53 protein has been demonstrated to be absent or mutated in approximately one out of two malignancies. It is known that p53-wt cancers have a better prognosis compared to p53-mut cancers. Our data are not in contradiction with this notion. Although both mutated and wild type p53 antibodies can be detected in cancer patients, their role is still controversial and a matter for debate. Recently, a few studies have reported that these antibodies are statistically associated with the survival of patients diagnosed with different malignancies. To the best of our knowledge, our meta-analysis is original and is the first study gathering p53 (wild type/mutated) antibody data generated from 1993 thus far. Overall, the investigation includes 12 studies and a total of 2094 patients.

## 4. Materials and Methods

The studies were identified according to the following inclusion criteria: (1) participants with cancer; (2) outcome results expressed in relation to the presence of a p53 antibody; (3) a primary outcome (disease-free survival, overall survival or progression-free survival) expressed as hazard ratio (HR). The following exclusion criteria were used: (1) insufficient data available to evaluate outcomes; (2) animal studies; (3) studies with less than 10 participants.

Two independent researchers revised the included studies, and all potential disputes that could have arisen were evaluated with the corresponding author.

The summary estimates were generated using a fixed-effect model (Mantel–Haenszel method) [27] or a random-effect model (DerSimonian–Laird method) [28] depending on the absence or presence of heterogeneity (I^2^). A subgroup analysis was performed to highlight any differences between studies in terms of Overall Survival (OS), Disease-Free Survival (DFS), Progression-Free Survival (PFS), as summarized in Table 1.

When we used the keywords “p53 antibodies in early cancer“, p53 antibodies in metastatic cancer”, “p53 antibodies impact on cancer progression”, the PubMed search yielded 1375 potentially relevant articles. Studies as duplicates, animal studies, cellular studies, or letters to the editor or reviews were excluded. After viewing the titles and abstracts, the full texts of 34 studies were retrieved and 12 studies [10,11,12,13,14,15,16,17,18,19,20,21] were included in the analysis because they had the hazard ratio available for survivals (Table 1 and Table 2) as summarized in the flow chart of Figure 1.

## 5. Conclusions

**Figure 4:**
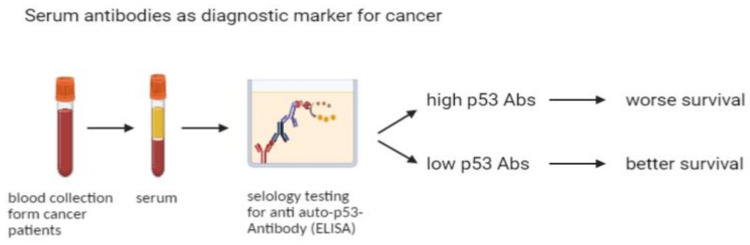
Schematic representation of the significance of serological biomarker p53 antibodies (p53Abs) in prediction of cancer survival.

We observed that serum antibodies generated in the blood of cancer patients against p53 (and mostly p53-wt) were deleterious. Given the straightforward detection in blood of p53 antibodies as a biomarker for cancer survival, as summarized in a simple workflow in Figure 4, these antibodies, together with other biomarkers, potentially constitute a valid method for prediction of cancer patients’ survival outcomes. The correlation could also play an important role for targeted therapies involving a cancer-suppressing p53 pathway.

## 6. Competing Interests

The authors have no relevant affiliations or financial involvement with any organization or entity with a financial interest in, or financial conflict with, the subject matter or materials discussed in the manuscript. This includes employment, consultancies, honoraria, stock ownership or options, expert testimony, grants or patents received or pending, or royalties. No writing assistance was utilized in the production of this manuscript.

## Figures and Tables

**Table 1:** Clinical investigations of p53-wt antibodies in cancer. Main characteristics of clinical investigations for prognostic evaluation of serum p53-wt antibodies in cancer patients.

Study Reference	Patients	Methods	Inclusion/Exclusion Criteria	Intervention	Follow-Up Time	Prognostic Value of s-p53-Abs	Type of Study
[10]	76 patients with transitional urinary bladder cell carcinoma	S-p53-Abs ELISA. Antibodies for p53-wt 184 CRC patients	Inclusion: transitional cell urinary bladder cancer Exclusion: secondary organ cancer; immunodeficiency state; ages over 90; other urinary bladder tumors.	Surgery (TUR) Surgery + chemotherapy + radiotherapy (advanced stage)	34 months	There was an association between the presence of s-p53-Abs and tumor p53 gene overexpression (*p =* 0.001).	Prospective
[11]	184 CRC patients. Dukes’ stage: A (*n* = 31); B (*n =* 84); C (*n =* 41); D (*n* = 28)	S-p53-Abs ELISA. Antibodies for p53-wt 184 CRC patients	Inclusion: primary colon cancer	Routine Biopsy Surgery	96 months	p53-Abs correlated with shorter survival (*p =* 0.02).	Retrospective
[12]	170 CRC patients	S-p53Ab, CEA ELISA. Antibody for p53-wt	Inclusion: primary colon cancer Exclusion: previous radiotherapy or chemotherapy	Surgery (resected tumor specimen)	93.6 months (median value)	Positivity for s-p53Ab in CRC did not correlate with overall survival. Kaplan–Meier analysis revealed significant differences between patients with elevated s-p53Ab and CEA and those with elevated levels of either one or neither of these factors (*p* < 0.001).	Retrospective
[13]	208 GC patients	S-p53Ab Detected with anti-p53 detection kit MESACUP anti-p53 Test Antibody for p53-wt	Inclusion: Histologically confirmed GC Exclusion: previously chemotherapy, radiotherapy and those who died within 30 days after surgery	Surgery	34 months	Did not observe any significant correlation between S-p53Ab in GC and overall survival (hazard ratio (HR) = 2.052; 95% confidence interval CI) = 0.891–4.726; *p =* 0.091). Conversely, Cox regression analysis revealed that a high level of CA19-9 was an independent prognostic factor for GC (hazard ratio (HR) = 3.864; 95% confidence interval (CI) = 1.248–11.959; *p =* 0.019).	Retrospective
[14]	231 SCLC patients	S-p53-Abs ELISA. Antibodies for p53-wt	Inclusion: primary SCLC	Surgery Chemotherapy (227 out of 231 patients)	3 months (at least)	High levels of p53-Abs correlated with worse survival prospects compared to patients with lower levels of the antibodies (*p* = 0.02).	Retrospective
[15]	80 HCC patients	S-p53-Abs ELISA. Antibodies for p53-wt	Inclusion: Cytohistological of AFP level-based diagnosis of HCC	Percutaneous injection (21) Surgery (15) Radiofrequency interstitial ablation (10) Chemotherapy (4) TACE (8) Combinational treatment (5) No treatment (17)	36 months	Anti-p53 was not useful as a prognostic factor.	Retrospective
[16]	244 CRC patients	CEA, CA19-9, S-P53Ab Antibody for p53-wt	Inclusion: preoperative CEA, CA-19 and S-P53Ab. Primary tumor diagnosis	Surgery (colectomy plus lymph nodes dissection) Chemotherapy (in case of CRC recurrence)	33.8 months (median)	S-P53Ab had no power to predict the prognosis (*p* = 0.786). Combined CEA and CA19-9 positivity was an exclusive independent prognostic factor (*p* = 0.034).	Retrospective
[17]	97 SCLC patients	S-p53-Abs ELISA. Antibodies for p53-wt	Inclusion: newly and proven diagnosed lung cancer	Bronchial biopsy Chemotherapy (cisplatin, etoposide, doxorubicin, cyclophosphamide) Radiotherapy for those with brain metastasis	18.1 months (median)	Patients with limited-stage SCLC and p53-Ab had a median survival time of 10 months, whereas limited-stage SCLC patients without p53-Ab had a 17-month median survival time (*p* = 0.014).	Prospective
[18]	133 esophageal squamous cell carcinoma (ESCC) patients	S-p53Ab, SCC-Ag, CEA Antibody for p53-wt	Inclusion: histologically confirmed ESCC Exclusion: patients who died after 30 days after treatment and those who had preoperative radiotherapy	Surgery	36 months (median)	S-p53Ab was detected in 39.1% (52 out of 133) of patients with ESCC, including 40.0% (20 out of 50) of patients with early-stage ESCC (*p* = 0.009)	Retrospective
[22]	201 lung cancer patients	S-p53 antibodies by ELISA	Inclusion: Primary lung cancer	Surgery Chemotherapy (Stage IIIB and IV) Radiotherapy (if required)	63 months	Patients with lower levels of p53Abs survived significantly longer than patients with higher levels of p53Abs (*p =* 0.049).	Retrospective
[19]	1487 esophageal squamous cell carcinoma	S-p53 antibodies by ELISA	Inclusion: radical surgery with no neoadjuvant treatment	Esophagectomy	42 months (median)	s-p53-Ab positive status was not significantly associated with poor overall survival	Retrospective
[20]	160 hepatocellular carcinoma	Six hepatocellular carcinoma-associated antigens, including Sui1, p62, RalA, p53, NY-ESO-1, and c-myc antibodies by ELISA (TAA Panel)	Inclusion: histologically proven HCC Exclusion: coexisting or metachronous cancer within 5 disease-free years	Surgery	60 months	The positivity for the TAA panel was independently associated with poor prognosis (*p* = 0.030)	Retrospective
[21]	72 gastric cancers	S-p53 antibodies by ELISA	Inclusion: primary gastric cancer Exclusion: previous chemotherapy; coexisting cancer	Surgery	32 months (median)	Overall survival was not associated with the antibodies	Retrospective
[23]	105 esophageal squamous cell carcinoma	S-p53 antibodies by ELISA	Inclusion: primary esophageal squamous cell carcinoma Exclusion: metastatic disease; neoadjuvant therapy	Surgery	35 months (median)	While seropositive patients did not demonstrate significant poor overall survival, high-titer patients demonstrated significant poor overall survival based on the multivariate analysis (*p* < 0.001).	Retrospective

Abbreviations: CRC, Colorectal Carcinoma; GC, Gastric Cancer; SCLC, Small Cell Lung Carcinoma; HCC, Hepatocellular Carcinoma; TACE, chemoembolization with epidoxorubicin and lipiodol; TUR, Transurethral Resection of the Tumor.

**Table 2:** Clinical investigations of p53-mut antibodies in cancer. Main characteristics of clinical investigations for prognostic evaluation of serum p53-mut antibodies in cancer patients.

Study Reference	Patients	Methods	Prognostic Value of s-p53-Abs	Type of Study	Inference
[24]	111 gastric carcinoma patients	S-p53-Abs Levels of p53-mut were determined with a selective, quantitative ELISA kit	The survival time of serum-positive patients was significantly longer than that of patients with low/negative serum levels, with a survival rate of 41.2% and 14.9%, respectively, over 48 months (*p* < 0.05).	Retrospective	Significant correlation seen between levels of S-p53-mut Abs and patient survival rate
[25]	104 ovarian cancer patients	S-p53-Abs ELISA. Antibodies against p53K132Q (c.394A > C).	Overall survival (OS) was significantly higher for patients with antibodies to mutant p53 when compared with patients without p53 antibodies (*p* = 0.01).	Retrospective	OS is significantly increased in advanced stage ovarian cancer patients with antibodies to p53
[17]	134 lung cancer patients	S-p53-Abs by Immunofluorescence. Antibodies against p53 R273H (c.818G > A) by ELISA.	Presence of anti-p53 autoantibodies is almost exclusively linked to the presence of malignant disease.	Retrospective	Presence of anti-p53 Abs had a significant correlation with shorter survival in NSCLC.
[26]	50 BC patients	S-p53-Abs ELISA. Antibodies against p53R273H (c.818G > A).	s-p53-Abs were higher in BC patients with high risk vs. patients with low risk. The difference was not statistically significant (*p =* 0.15).	Retrospective	Presence of s-p53-Abs showed higher risk for BC patients.

## Data Availability

All relevant data are within the paper.

## Sample Availability

Samples of the compounds are not available from the authors.
